# Impact of second victim distress on healthcare professionals' intent to leave, absenteeism and resilience: A mediation model of organizational support

**DOI:** 10.1111/jan.16291

**Published:** 2024-06-19

**Authors:** Sanu Mahat, Helena Lehmusto, Anne Marie Rafferty, Katri Vehviläinen‐Julkunen, Santtu Mikkonen, Marja Härkänen

**Affiliations:** ^1^ Department of Nursing Science University of Eastern Finland Kuopio Finland; ^2^ Jorvi Hospital Helsinki University Hospital Pharmacy Espoo Finland; ^3^ Florence Nightingale Faculty of Nursing, Midwifery & Palliative Care King's College London London UK; ^4^ Department of Environmental and Biological Sciences, Faculty of Science, Forestry and Technology University of Eastern Finland Kuopio Finland; ^5^ Wellbeing Services County of North Savo, Research Centre for Nursing Science and Social and Health Management Kuopio University Hospital Kuopio Finland

**Keywords:** absenteeism, distress, healthcare professionals, medication error, organizational support, resilience, second victim, turnover intention

## Abstract

**Aims:**

To examine the relationship between the second victim distress and outcome variables, specifically: ‘turnover intentions, absenteeism and resilience’. Furthermore, this study also assessed how organizational support mediates the relationship between second victim distress and outcome variables.

**Design:**

Cross‐sectional survey.

**Methods:**

A cross‐sectional survey study using regression and mediation analysis with bootstrapping was conducted among (*n* = 149) healthcare professionals in two university hospitals in Finland from September 2022 to April 2023 during different time periods. The Finnish version of the revised Second Victim Experience and Support Tool (FI‐SVEST‐R) was used to assess second victim distress, level of organizational support and related outcomes.

**Results:**

Psychological distress was the most frequently experienced form of reported second victim distress, and institutional support was the lowest perceived form of support by healthcare professionals. The study found second victim distress to have a significant association with work‐related outcomes: turnover intention and absenteeism. However, no significant relationship was found with resilience. Mediation models with organizational support revealed a partially mediated relationship between second victim distress and work‐related outcomes.

**Conclusions:**

The findings from this study indicate that second victim experiences if not adequately addressed can lead to negative work‐related outcomes such as increased job turnover and absenteeism. Such outcomes not only affect healthcare professionals but can also have a cascading effect on the quality of care. However, the mediating effect of organizational support suggests that if comprehensive support is provided, it is possible to mitigate the negative impact of the second victim phenomenon.

**Impact:**

Raising awareness regarding the second victim phenomenon, promoting a culture of safety and shifting the paradigm from a blame to just culture helps in identifying the system flaws thus improving both patient and provider safety.

**Reporting Method:**

The study adheres to the STROBE reporting guidelines.

**Patient or Public Contribution:**

No patient or public contribution.

## 1 INTRODUCTION

Harm to the patient during health care has been considered a leading cause of death and disability globally, most of which are avoidable. Medication‐related harm along with other therapeutic care‐related harm account for nearly half of all avoidable harm in health care (Panagioti et al., 2019). A report on the prevalence and burden of medication errors (MEs) in England estimated 237 million MEs to have occurred per annum (Elliott et al., 2018). Patient safety events like MEs can lead to three types of victims: the patient and his/her family as the first victim, healthcare providers as the second victim (SV) and the healthcare organization as the third victim. Healthcare providers who are directly involved in adverse patient safety events and remain traumatized are identified as ‘SVs’ (Wu, 2000). After 2000, the term ‘SV’ was deployed widely (Wu, 2000). This however prompted a backlash for some arguing that it was a way for healthcare professionals (HCPs) to deny their accountability for the errors that have occurred (Clarkson et al., 2019; Tumelty, 2021). Subsequent research has clarified the use of the term SV as a useful way to draw the attention of policymakers to the seriousness of the issue (Wu et al., 2020).

## 2 BACKGROUND

The research related to SV has been advancing in the past decade with a concrete definition of the second victim phenomenon (SVP) (Scott et al., 2009). An instrument called the Second Victim Experience and Support Tool (SVEST) has been devised to measure the severity of SVP (Burlison et al., 2017). Research has found SVP to be quite common with approximately half of the HCPs being a SV at least once in their career (Seys et al., 2013; White & Delacroix, 2020). A systematic review focusing on SVP anticipated the prevalence of SVP to vary between 10.4% and 43.3% in healthcare settings (Seys et al., 2013). The SV prevalence has been measured and reported in various countries. Previous research on the prevalence of SVP has shown that in an Austrian healthcare institute, the prevalence was found to be 43% (Krommer et al., 2023). A German study conducted among nurses working in various settings found that 60% of nurses experienced SVP at some point with a 12‐month prevalence of 49% (Strametz, Fendel, et al., 2021). Similarly, among German physicians, 59% reported having experienced SVP at least once in their career with a 12‐month prevalence of 35% (Strametz, Koch, et al., 2021). A study conducted among intensive care unit nurses found 67% of participants to have reported psychological distress after adverse events (Kappes et al., 2023). These adverse events can have a negative impact on the health and functional ability of HCPs and reduce their coping abilities (Busch et al., 2020a; Kappes et al., 2021). HCPs can experience various physical and psychological symptoms such as depression, anxiety, shame, anger, remorse and troubled memories related to the event which often turn out to be nightmares preventing them from having a sound sleep (Busch et al., 2020b). This might further lead HCPs to doubt their professional self‐efficacy (Mahat et al., 2022) and jeopardize the safety of patients due to poor quality of care and defensive behaviours (Liukka et al., 2020; Panella et al., 2016). Previous research revealed that HCPs after MEs can make far‐reaching decisions such as requesting for transfer, taking time off from work or abandoning their profession if the support structures are not in place (Mira et al., 2015). Hence, the consequences of SVP can be long‐lasting and life changing. A counter argument has suggested that distress symptoms experienced by SVs might promote resilience and improve mental health for HCPs (Winning et al., 2021). Previous research has shown that support received right after MEs can be effective in mitigating SVP and help in the recovery of SVs (Mahat et al., 2022; Scott et al., 2010).

If adequate support exists, this could help develop resilience and promote positive progress in their work by learning from errors. Resilience has been defined as an innate trait that enables a person to overcome suffering, learn from painful experiences and become stronger (Mokline et al., 2021). A resilient HCP responds to stressful events with an adaptive approach to achieve healthcare goals with less physical and psychological burden (Parks‐Savage et al., 2018). Previous research has shown psychologically resilient nurses to have better‐coping abilities and to have experienced more positive emotions (Guo et al., 2018). Resilience on the other hand can be considered as an attribute of the system and not of individuals alone. Hence, integrating support in the systems is essential to enable HCPs to operate in a psychologically safe environment (Sheikhrabori et al., 2022). Variable measures have been identified as helpful in handling SVP, which includes personal and organizational strategies. Support for SVs is crucial not just for their personal well‐being but also for maintaining quality of care, thereby preventing further negative outcomes (Ullström et al., 2014). Peer group support or support from immediate colleagues has been found to be more effective in mitigating SVP (Busch et al., 2020a; Kappes et al., 2021). Additionally, healthcare institutions that have a non‐punitive and non‐judgmental environment are essential for significantly reducing the intensity of SVP (Zhang et al., 2019). In contrast, poor organizational support has been found to generate negative and potentially devastating repercussions among HCPs (Wu et al., 2020).

Research related to SVP is abundant (Coughlan et al., 2017) and numerous support strategies have been identified, one of the first of which was Resilience in Stressful Events (RISE) from Johns Hopkins Hospital (Edrees et al., 2016), and a peer support program at Brigham and Women's Hospital known as Centre for Professionalism and Peer Support (CPPR) (Shapiro et al., 2014). However, there is a gap in research in Finland regarding SVP and the support HCPs receive from the organization following adverse events including MEs. As far as we know, this is the first study to use the Finnish version of the revised SVEST tool named as FI‐SVEST‐R to explore the relationship between SV triggers and three outcome variables, two work‐related negative outcomes: ‘turnover intentions and absenteeism’ and one positive outcome: ‘resilience’ in Finland. Within Finland, there have been few studies conducted on SVP one of which was based on nurse managers' perception of support interventions for nurses who are SVs. This study revealed the challenges and support received by nurse managers while managing tasks related to second victim support and interventions (Järvisalo et al., 2023). However, no studies have investigated the distress faced by HCPs following MEs including its impact on their professional life and HCPs' perception regarding the available support resources. We focused only on ME‐related SVP given that MEs are highly common in an in‐hospital setting (Härkänen et al., 2018). This study is the first we know to describe the distress symptoms HCPs have faced following MEs and link it to the adequacy of support they have received in the aftermath of MEs.

## 3 THE STUDY

### 3.1 Aims

This study aimed to examine the relationship between second victim distress and outcome variables which are as follows: ‘turnover intentions and absenteeism’ and ‘resilience’. It also assessed how organizational support mediates the relationship between second victim distress and outcome variables.

### 3.2 Hypothesis

Based on these aims, the study proposes the following hypothesis:

HCPs experiencing SV distress who receive inadequate organizational support will report increased intent to leave their profession, higher rates of absenteeism and lower levels of resilience, compared to those who receive adequate support.

## 4 METHODS

### 4.1 Study design

This study used a quantitative cross‐sectional survey design. The Strengthening the Reporting of Observational studies in Epidemiology (STROBE) guidelines (Supplementary file S1) were used to report the study (von Elm et al., 2007).

### 4.2 Study setting and participants

The study was conducted in two major university hospitals in Finland (Eastern and Southern Finland) at different time points between September 2022 and May 2023. Nurses and physicians were selected as the study population because of their direct involvement in patient care and the medication administration process. Of 498 opened survey links, 163 (32.7%) nurses and physicians participated in the survey. At the time of data review, 14 responses were excluded as they were incomplete, and 149 responses were included for final analysis.

### 4.3 Procedures and measures

A convenience sampling approach was adopted to recruit potential respondents. Nurses and physicians involved in at least one medication error incident were invited to take part in the research. The recruitment of participants was done via email invitation with the help of a contact person at each hospital. The invitation was sent to approximately 4000 potential participants. The invitation email included a description of the survey, information to participants and a survey link. The survey was created using Webropol software (V3.0; Webropol Oy, Helsinki, Finland), a secure web‐based application used to collect data.

### 4.4 Inclusion criteria

The inclusion criteria for participants were (1) being a nurse or physician, (2) working in any of the two hospitals, (3) being involved in work related to the medication process and (4) having experienced MEs at least once in their working career.

### 4.5 Study instrument and measurement of the variables

The original SVEST scale was first developed by Burlison et al. (2017) and later revised by Winning et al. (2021) named as SVEST‐R. This tool has been translated from the original English version into different other languages since its development such as in Germany (Strametz et al., 2022) and Malaysia (Mohd Kamaruzaman et al., 2022b). The original SVEST consisted of seven dimensions with two negative outcome variables. Seven dimensions cover: psychological distress (4 items), physical distress (4 items), colleague support (4 items), supervisor support (4 items), institutional support (3 items), non‐work‐related support (2 items) and professional self‐efficacy (4 items). Turnover intentions (2 items) and absenteeism (2 items) are the two negative outcome variables. All the items are closed statements based on a five‐point Likert scale ranging from 1 (strongly disagree) to 5 (strongly agree). Higher scores indicate a higher prevalence of SV responses, insufficient support resources and a higher magnitude of negative work outcomes.

The revised version of SVEST (SVEST‐R) consists of a 35‐item questionnaire, in which the non‐work‐related support dimension was omitted, and a positive outcome dimension ‘resilience’ was added. SVEST‐R demonstrated good construct validity (chi‐square test x^2^ = 1555, degree of freedom [DOF] = 524, root mean square error of approximation (RMSEA) = .079, comparative fit index [CFI] = 0.821 and standardized root mean squared residual [SRMR] = 0.091). Cronbach's alpha ranged from .66 for colleague support to .86 for physical distress with factor loadings of all items ranging from .42 to .92 (Winning et al., 2021).

#### 4.5.1 Translation and cultural adaptation to Finnish context

The last author of this paper contacted the original author of the SVEST‐R via email to obtain permission and authorization for its translation into the Finnish language. After receiving permission from the original author, all the standardized procedures of translation and cultural adaptation were followed. Forward translation of the tool was performed by two researchers, and back translation was performed by a professional translator expert in medical English and Finnish language. An expert panel of eight members (experts in patient safety, quality, clinical area, teaching, research and health care) was recruited to review the translation process. Few wordings and sentence structures were revised with the suggestion of expert panel for its adaptability to Finnish context (World Health Organization, 2024b). This instrument which is now called FI‐SVEST‐R is a validated instrument which consists of 35 items/statements to which respondents indicate the extent of their agreement (5‐point Likert scale: 1 = strongly disagree, 5 = strongly agree) with each statement relating to their own experiences of medication incidents and support strategies meaning that Likert scale scores >3 means agreement and <3 means disagreement. For support dimensions: colleague support, supervisor support and institutional support, higher scores on the percentage of agreement represent lower perceived support.

##### Predictor variable

For this study, 12 items assessing the components of psychological distress, physical distress and reduced professional self‐efficacy (4 items each) were used to measure second victim distress symptoms. Mean was computed for responses related to these 12 items to create a single second victim distress dimension.

##### Mediator variable

Similarly, 11 items assessing the perceived support received from colleagues, supervisors (team leaders and managers) and institutions were combined, and a single dimension named organizational support was created by computing means of responses for these 11 items.

##### Dependent variables

For three outcome variables, four items measured turnover intentions, three items measured absenteeism related to medication errors, and four items were used to measure resilience developed after medication errors. The reverse‐coded items were re‐coded in SPSS before running the analysis.

### 4.6 Data collection

The researchers sent a web link along with a participant information sheet to the potential participants (nurses and physicians) via the contact person identified through collaborative research in respective institutions. Webropol was used to create the online survey. The first page of the survey included informed consent and background information required from the participants. If participants did not want to give informed consent and select ‘No’, the survey automatically directed them to the ‘Thank you’ page. Those who gave their informed consent to participate could move forward and complete the survey anonymously.

### 4.7 Data analysis

For demographic variables, descriptive statistics and Spearman's rank‐order correlations were computed. Analysis of covariance (ANCOVA) was performed to account for multivariable relationships between predictor and outcome variables. Due to the high correlation between age and work experience, they could not be used in the model at the same time without problems with multicollinearity (Table 2). Therefore, only work experience was used in the models.

To evaluate mediation effects, we employed two distinct methodologies. Initially, we applied the four‐step technique proposed by Barron and Kenny, which involved conducting a series of regression analyses to scrutinize the significance of coefficients at each phase (Baron & Kenny, 1986). Subsequently, we adopted the bootstrapping method to assess the indirect impact of second victim distress on outcome variables (turnover intentions, absenteeism and resilience), utilizing 5000 bootstrap samples. We used bootstrapping technique to support the mediation as it helps to quantify, and account for, the uncertainties in the analysis. These analyses were performed using IBM© SPSS© version 27 alongside macro by Preacher and Hayes (Preacher & Hayes, 2004).

### 4.8 Ethical considerations

This study was approved by the University of Eastern Finland ethics committee (decision number: 36/2022) in October 2022, and a research permit was obtained from both university hospitals (decision number: 50UL043) in February 2023. The anonymity of the participants was assured, and the data were only available for authors. Informed consent was obtained from each participant.

## 5 RESULTS

### 5.1 Sample characteristics

Table 1 shows the descriptive statistics for the demographic variables. The descriptive statistics for the study variables and the correlation among the study variables can be found in Table 2. The majority of respondents were female (91.9%) and were working as registered nurses (90%). About one‐third (31.5%) of respondents had work experience of more than 21 years and 21.5% of respondents had work experience of 1–5 years.

**TABLE 1:** Socio‐demographic variables.

Socio‐demographic variables	*n* (%)
Age (years)	
<25	3 (2)
25–35	40 (26.8)
36–45	37 (24.8)
46–55	45 (30.2)
56–65	23 (15.4)
>66	1 (0.7)
Gender	
Male	10 (6.7)
Female	137 (91.9)
Other	2 (1.4)
Profession	
Nurses	139 (93.3)
Physician	10 (6.7)
Work experience categories (years)	
1–5	32 (21.5)
6–10	28 (18.8)
11–15	28 (18.8)
16–20	14 (9.4)
>21	47 (31.5)

### 5.2 Descriptive statistics

Demographic variables ‘work experience (–.16, *p* < .05)’ and ‘age (–.21, *p* < .05)’ were associated negatively and significantly with study variable turnover intentions which exhibit that HCPs having greater amounts of work experience are less likely to leave their job. Similarly, younger age HCPs are more likely to leave their job compared to their older counterparts. Among study variables, the predictor variable second victim distress and the mediating variable organizational support had statistically significant strong positive correlation with two outcome variables (turnover intentions and absenteeism, *p* < .01). Similarly, the outcome variable ‘turnover intention’ was moderately positively correlated with resilience. However, there was no significant correlation between other variables and the third outcome variable ‘resilience’ (Table 2).

**TABLE 2:** Mean (standard deviation), Spearman rank‐order correlations and Cronbach's alpha for demographic and study variables.

Variable	Mean (SD)	Years of work experience	Age	Second victim distress	Organizational support	Turnover intentions	Absenteeism	Resilience
Demographic variables								
Years of work experience	15.51 (10.5)	–						
Age	43.3 (10.8)	.80**	–					
Study variables								
Second victim distress	2.89 (0.83)	−.14	−.11	(.891)				
Organizational support	2.48 (0.63)	−.11	−.11	.47**	(.834)			
Turnover intentions	2.26 (1.07)	−.16*	−.21*	.66**	.54**	(.898)		
Absenteeism	1.77 (.86)	−.04	−.07	.54**	.48**	.63**	(.793)	
Resilience	2.28 (.75)	−.05	−.07	.08	.09	.25**	.09	(.762)

*Note*: () is Cronbach's alpha coefficient for study variables.

Abbreviation: SD, standard deviation.

* Significance at level *p* < .05;

** Significance at level *p* < .01.

### 5.3 Second victim experience and support

Based on the responses received from HCPs (Table 3), 36.2% and 10.7% participants responded as suffering from psychological distress and physical distress after MEs, respectively, and 7.4% of participants reported to have experienced reduced professional self‐efficacy. Similarly, regarding support, 35.8% of participants perceived institutional support as poor, and 3.4% and 4% of participants perceived colleague support and supervisor support as poor, respectively. Also, 10.1% of HCPs agreed on having thoughts about leaving the profession, 3.4% agreed on taking some time off and only 2.7% agreed on developing resilience after encountering MEs.

**TABLE 3:** Agreement percentages for dimensions of FI‐SVEST‐R (*n* = 149).

Dimensions	Agreement (%)
Psychological distress	36.2
Physical distress	10.7
Professional self‐efficacy (reduced)	7.4
Colleague support	3.4
Supervisor support	4.0
Institutional support	35.8
Turnover intentions	10.1
Absenteeism	3.4
Resilience	2.7

*Note*: Higher support agreement percentages denote healthcare professionals' perception of inadequate support determined based on Likert scale scores (>3 means agree and <3 means disagree).

### 5.4 Association between HCPs' work experience, second victim distress, work‐related outcomes and resilience

Regression analysis was conducted using ANCOVA to compare the effect on HCPs' turnover intentions, absenteeism and resilience across different categories of length of work experience while adjusting for the predictor variable second victim distress. Regarding the influence of work experience on outcomes (turnover intentions, absenteeism and resilience), after controlling for second victim distress, no significant effects were observed across the different categories of work experience (Table 4). Results revealed a significant relationship among predictor variable ‘second victim distress’ and outcome variables ‘turnover intention (*B* = 0.786, *p* < .001) and absenteeism (*B* = 0.557, *p* < .001)’ when controlling for the effect of work experience. However, no significant relation was found between the predictor and outcome variable ‘resilience (*B* = –0.015, *p* = .078)’.

**TABLE 4:** Regression analysis results of second victim distress and length of work experience predicting turnover intention, absenteeism and resilience.

	Turnover intentions				Absenteeism				Resilience			
	*B*	SE	*p*	Partial *η* ^2^	*B*	SE	*p*	Partial *η* ^2^	*B*	SE	*p*	Partial *η* ^2^
Second victim distress												
	0.786	0.087	<.001	0.363	0.557	0.078	<.001	0.265	−0.015	0.078	.851	0.000
Categories of work experience in years												
1–5	0.228	0.194	.243	0.010	−0.036	0.173	.835	0.000	0.145	0.175	.409	0.005
6–10	0.351	0.205	.089	0.020	−0.091	0.183	.618	0.002	0.175	0.185	.346	0.006
11–15	0.111	0.209	.596	0.002	−0.329	0.186	.079	0.021	−0.037	0.188	.845	0.000
16–20	0.029	0.260	.911	0.000	0.014	0.232	.953	0.000	0.006	0.234	.981	0.000
>21	0a				0a				0a			

*Note*: The reference category (>21 years) is indicated as having its parameter set to Zero (0a) because it is redundant. Partial eta squared (partial *η* ^2^) represents the proportion of total variance in outcome variables attributed to each predictor variable (values of partial *η* ^2^ range from 0 to 1, where 0 = no effect, 1 = perfect effect, 0.01 = small effect, 0.06 = medium effect, 0.14 = large effects).

Abbreviation: B, unstandardized regression coefficient; SE, standard error.

### 5.5 Mediation analysis results

**FIGURE 1:**
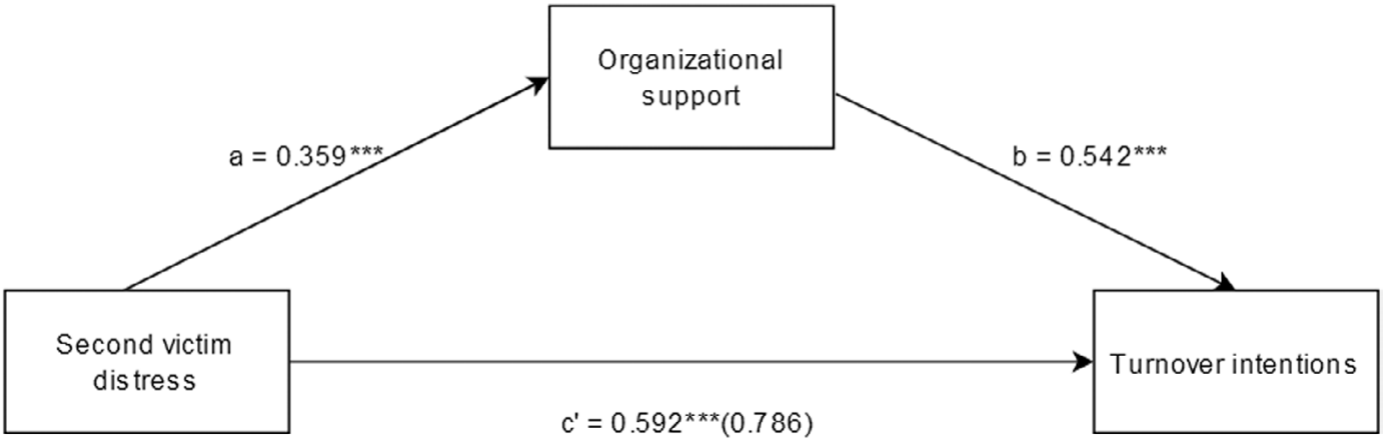
The mediation model between second victim distress, organizational support and turnover intentions. ***p < .001. The value in parentheses () refers to the unstandardized regression coefficient between second victim distress and turnover intentions while controlling for organizational support.

**FIGURE 2:**
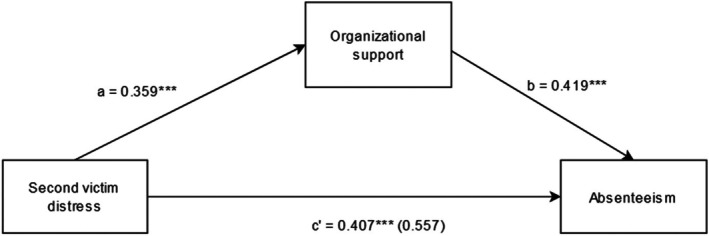
The mediation model between second victim distress, organizational support and absenteeism. ***p < .001. The value in parentheses () refers to the unstandardized regression coefficient between second victim distress and absenteeism while controlling for organizational support.

This study assessed the mediating role of healthcare professional's perceived organizational support on the relationship between second victim distress symptoms and two work‐related outcomes (turnover intention and absenteeism) and resilience. Results of mediation analyses confirmed that organizational support partially mediated the relationship between second victim distress symptoms and work‐related outcomes (Figures 1, 2). Since both ‘a‐path’ and ‘b‐path’ were significant in both work outcome models (Figures 1, 2), mediation analyses were tested using the bootstrapping method with bias‐corrected confidence estimates (MacKinnon et al., 2004; Preacher & Hayes, 2004). The 95% confidence interval of the indirect effects was obtained using 5000 bootstrap resamples (Preacher & Hayes, 2008). When controlled for the demographic variables, none of the variables were significant contributors to all three models assessing turnover intention, absenteeism and resilience as the outcome variables.

The results of the mediation analyses revealed a significant indirect effect of the impact of SV distress symptoms on turnover intentions (*B* = 0.195, *t* = 6.472) and work absenteeism (*B* = 0.150, *t* = 4.907) verifying the aforesaid study hypothesis. Furthermore, the direct effect of SV distress symptoms on work‐related outcomes in the presence of the mediator (organizational support) was also found to be significant: turnover intention (*B* = 0.592, *p* < .001) and absenteeism (*B* = 0.407, *p* < .001). Hence, organizational support partially mediated the relationship between the SV distress symptoms and work‐related outcomes. The partial mediation effect suggests that perceived low level of organizational support may further deteriorate SV distress symptoms and increase the desire to quit or take time off from work (Table 5).

**TABLE 5:** Mediation analysis summary using bootstrapping in three different outcome models.

	Paths		*B*	*p*‐value	Conclusion
Role of organizational support between second victim distress and turnover intention	a path	Second victim distress → organizational support	.359	<.001	Partial mediation
	b path	Organizational support → turnover intention	.542	<.001	
	c path (total effect)	Second victim distress → turnover intention	.786	<.001	
	c’ path (direct effect)	Second victim distress → turnover intention	.592	<.001	
	a × b path (indirect effect via organizational support)	Second victim distress → turnover intention	.195	^a^	
Role of organizational support between second victim distress and absenteeism	a path	Second victim distress → organizational support	.359	<.001	Partial mediation
	b path	Organizational support → absenteeism	.419	<.001	
	c path (total effect)	Second victim distress → absenteeism	.557	<.001	
	c’ path (direct effect)	Second victim distress → absenteeism	.407	<.001	
	a × b path (indirect effect via organizational support)	Second victim distress → absenteeism	.150	^b^	
Role of organizational support between second victim distress and resilience	a path	Second victim distress → organizational support	.359	<.001	No relationship, No mediation
	b path	Organizational support → resilience	.194	.083	
	c path (total effect)	Second victim distress → resilience	−.015	.845	
	c’ path (direct effect)	Second victim distress → resilience	−.084	.337	
	a × b path (indirect effect via organizational support)	Second victim distress → resilience	.069	^c^	

*Note*: *B* is beta coefficient. If the 95% CI does not contain zero, a significant indirect effect via mediators between dependent and independent variables was determined.

^a^ 95% confidence interval (CI) from 0.0939 to 0.2935.

^b^ 95% CI from 0.0569 to 0.2464.

^c^ 95% CI from −0.0319 to 0.1663.

However, in the case of the relationship between SV distress symptoms and resilience, no significant relationship was found either in the presence or absence of a mediating variable suggesting that an increase or decrease in SV distress has no effect on resilience among HCPs.

## 6 DISCUSSION

The present study aimed to highlight the existing relationship between SV distress and HCPs' intention to turnover, absenteeism and resilience after MEs. Furthermore, this study also investigated the perceived organizational support by HCPs after MEs and the impact of the support in ameliorating SV distress which will reduce negative outcomes (turnover intention and absenteeism) and increase resilience among HCPs.

Results from correlation analysis revealed a significantly higher correlation between age and duration of work experience of HCPs which can be easily understood because of the linear trajectory between these two variables. Therefore, only the duration of work experience was included in the later analysis.

Our study found that the highest agreement percentage recorded was for psychological distress than for physical distress after MEs which shows that psychological distress emerged as the most affected dimension among participants in this study. It is foreseeable that the dimension psychological distress received the highest scores of agreement as the storm of the negative emotions HCPs experience after MEs can last for a longer period (Mahat et al., 2022). This finding is consistent with other study findings investigating SVP (Brunelli et al., 2021; Draus et al., 2022; Mathebula et al., 2022). Similarly, the feeling of embarrassment after facing errors was found to be more prevalent among HCPs (Busch et al., 2020b).

HCPs in this study perceived inadequacy of support structures from the side of healthcare institutions expressing greater agreement with the lowest perceived support for the institutional support dimension followed by supervisor support. This finding is supported by previous studies where HCPs perceived limited institutional support after patient safety incidents (Mathebula et al., 2022). Previous studies have also found healthcare organizations focusing on the patient‐centred approach after adverse events, but having no systematized plans for HCPs who are equally facing emotional turmoil (Kappes et al., 2021). Healthcare institutions' strategies for quality healthcare must have a systematic support plan designed for SVs (Busch et al., 2021). The low agreement percentage of collegial support denotes that most of the HCPs found collegial support to be helpful and adequate after MEs. Similar findings have been reported in a previous study by Draus et al. (2022), where SVs asked for help from a colleague rather than contacting their supervisor for support after an adverse event.

Major findings of this study indicate a significant association between SV distress and turnover intentions showing that HCPs experiencing more SV distress are the ones having higher intentions to leave their job. This study also suggests that SV distress predicts HCPs' intentions to take time off or remain absent from their work. HCPs were found to consider the thought of exiting the healthcare profession as a repercussion of the distress they face due to MEs. These findings complement previous research findings reporting the effects of the SVP on HCPs' intent to leave and absenteeism (Burlison et al., 2021; Mohd Kamaruzaman et al., 2022a; Mok et al., 2020; Scott et al., 2009; Zhang et al., 2019). A positive correlation between SV distress and turnover intentions has been found by prior studies suggesting that an increase in SV‐related distress causes an increase in HCPs' turnover rate (Burlison et al., 2017; Finney et al., 2021; Rivera‐Chiauzzi et al., 2022).

Another major finding of this study was the mediating role of organizational support in explaining the relationship between SV distress and negative work outcomes: turnover intention and absenteeism. The relationships observed between second victim distress, turnover intentions and absenteeism were found to occur through a pathway characterized by perceived lower levels of support from the organization. Lack of organizational support or litigation has been found to exacerbate the SV distress symptoms among HCPs (Burlison et al., 2021). Furthermore, this might entail a significant impact regarding the loss of HCPs due to an increase in staff turnover rate and absenteeism (Burlison et al., 2021). The role of organizational support in alleviating SV distress associated with adverse events has been explained by previous studies which in turn reduces HCPs' intention to leave and take time off from the job (Burlison et al., 2021). Healthcare organizations thus play an important role in mitigating the SV distress symptoms by providing the required support. The greater the impact of the SVP, the lower HCPs have perceived as receiving support or other existent support measures (Brunelli et al., 2023). The intensity of negative work‐related outcomes was noticeably reduced when non‐judgmental and non‐punitive organizational support was given after adverse events (Van Gerven et al., 2014). Also, the existence of support protocols in an institution does not necessarily mean staff receiving proper and timely support. For HCPs to be aided by the support resources, a just culture is needed where it can be understood that HCPs are not solely to be blamed for adverse events (Ullström et al., 2014).

However, when examining the relationship between SV distress and another outcome resilience with or without considering the mediating role of organizational support, no significant association was found. This finding suggests that HCPs' ability to develop resilience neither depends upon the intensity of second victim distress they experience nor the level of perceived support. This finding contrasts with previous research which reported that resilience could be developed by providing mutual respect to HCPs experiencing psychological distress aftermath of adverse events (Robertson & Long, 2019). Resilience was found to be a protective factor for SVs (Xu et al., 2022) as higher psychological resilience enables nurses to face work challenges and cope effectively (Guo et al., 2018). Also, research on resilience after adverse events has found that HCPs might develop adaptive behaviours allowing them to frequently avoid making errors (Winning et al., 2021). Our study findings emphasize the need to understand how organizational cultures and practices contribute to or detract from resilience, rather than focusing solely on individual traits.

### 6.1 Limitations

A cross‐sectional survey was used to determine the impact of ME‐related SV distress on work‐related outcomes; therefore, it is difficult to determine how the impact of MEs has changed over time. Although the possibility of other confounding factors and limited sample size and power to analyse causality could not be denied, this study was successful in exploring SVP and perception of support after MEs among HCPs in Finland.

Even though attempts were made to recruit more male members and physicians for a diverse sample, the majority of the respondents were female and nurses. The reason behind most participants being nurses could be that the nurses may perceive that their input in recognizing the seriousness of SVP could influence policies or bring improvements in support strategies. Also, as the study focuses on ME‐related SVP, nurses are typically responsible for administering medications to the patients which places them directly at the point where errors most likely occur. Also, nurses are the first ones to notice and handle any adverse reactions caused due to MEs. Regarding the predominance of female participants, it could be because women make up a significant majority of the nursing workforce globally (World Health Organization, 2024a). The ongoing pandemic and safety concerns posed limitations on using onsite data collection; therefore, online platforms were used to reach participants which despite being cost‐effective and feasible hugely impacted the number of responses received. Furthermore, the persistent stigma related to shame, embarrassment, blame or judgmental culture might have served as a challenge for respondents to answer the survey even though their anonymity and confidentiality were assured. Although our measures are based on participants' self‐reported data, which can introduce biases, we have mitigated this concern by using instruments that have strong evidence of validity and reliability.

### 6.2 Future recommendations

Healthcare institutions implementing SV support programs need to publicize the usage of such programs among HCPs. Despite the availability of resources to aid SVs, the impact is negligible if HCPs are unaware of its accessibility and procedures to use these resources. A previous study has also reported that around 68.8% of SVs indicated a lack of awareness regarding hospital support resources (Draus et al., 2022). Furthermore, future research studies should aim at conducting intervention studies related to SVP, and more specialized SV support programs are needed globally. Similarly, future studies should also aim to investigate the causes leading to most MEs in Finnish healthcare institutions. Additionally, due to very less responses from physicians, this study could not examine how the responses differed between nurses and physicians, as these may differ on several factors such as the nature of ME, work climate and support. Therefore, future research should consider examining these differences to allow management to identify the needs of different HCPs.

## 7 CONCLUSION

Our findings underscore the necessity for healthcare organizations to recognize the significance of the SVP and to prioritize the implementation of structured support systems. This study also contributes valuable insights into the relationship between distress experienced by SVs, turnover intention, absenteeism and the crucial role of organizational support. The findings from this study warrant more research related to SVP in Finland among a diverse group of HCPs. As the support from healthcare institutions after MEs was deemed low by the respondents, this important finding highlights the need for system change and cultivating a supportive work environment for HCPs following MEs.

## 8 RELEVANCE TO CLINICAL PRACTICE

This study sheds light on the amount of psychological, physical and emotional distress HCPs can face aftermath of MEs. Understanding the impact of the SVP can promote the development of a blame‐free and just culture that prioritizes learning from errors rather than blaming the error makers. It ensures that healthcare systems should not only focus on patient safety but also on the well‐being of those who provide care. Recognizing HCPs as SVs emphasizes the need for support, thus encouraging a more constructive response to errors. In the presence of appropriate support, HCPs can develop resilience, improve coping strategies and enhance their professional competencies which in turn creates a more robust healthcare system and contributes to quality care. Healthcare institutions can mitigate negative work‐related outcomes like career exit and abandonment, turnover intentions and job dissatisfaction by offering structured support.

## AUTHOR CONTRIBUTIONS

All the authors made significant contributions in the conception and design of the study, acquisition of data, data analysis and interpretation of findings. Drafting of the manuscript was done by the corresponding author, statistical analysis was done with the help of a statistician who is also the author of this study. All the authors revised the manuscript critically and gave their final approval for the version of the manuscript to be submitted. S.M., H.L., AM.R., K.V‐J., S.M. and M.H. made substantial contributions to conception and design, or acquisition of data, or analysis and interpretation of data; S.M., AM.R., K.V‐J. and M.H. involved in drafting the manuscript or revising it critically for important intellectual content; S.M., H.L., AM.R., K.V‐J., S.M. and M.H. gave final approval of the version to be published. Each author should have participated sufficiently in the work to take public responsibility for appropriate portions of the content; S.M., H.L., AM.R., K.V‐J., S.M. and M.H. agreed to be accountable for all aspects of the work in ensuring that questions related to the accuracy or integrity of any part of the work are appropriately investigated and resolved.

## FUNDING INFORMATION

This research received no specific grant from any funding agency in the public, commercial or not‐for‐profit sectors.

## CONFLICT OF INTEREST STATEMENT

No conflict of interest has been declared by the authors.

## PEER REVIEW

The peer review history for this article is available at https://www.webofscience.com/api/gateway/wos/peer‐review/10.1111/jan.16291.

## Supporting information


Supplementary File S1:

## Data Availability

The data that support the findings of this study are available on request from the corresponding author. The data are not publicly available due to privacy or ethical restrictions.

## References

[jan16291-bib-0001] Baron, R. , & Kenny, D. A. (1986). The moderator‐mediator variable distinction in social psychological research: Conceptual, strategic, and statistical considerations. Journal of Personal and Social Psychology, 51(6), 1173–1182. 10.1037/0022-3514.51.6.1173 3806354

[jan16291-bib-0002] Brunelli, M. V. , Estrada, S. , & Celano, C. (2021). Cross‐cultural adaptation and psychometric evaluation of a second victim experience and support tool (SVEST). Journal of Patient Safety, 17(8), E1401–E1405. 10.1097/PTS.0000000000000497 29733300

[jan16291-bib-0003] Brunelli, M. V. , Silvina, E. , Constanza, C. , Bandriwskyj, C. , Riquelme, R. J. , Ortega, A. , Gonzalez, E. G. , Monserrat, I. E. , & Mesurado, B. (2023). Second victim experience and support from health professionals. Medicina, 83(6), 918–926. 38117711

[jan16291-bib-0004] Burlison, J. D. , Quillivan, R. R. , Scott, S. D. , Johnson, S. , & Hoffman, J. M. (2021). The effects of the second victim phenomenon on work‐related outcomes: Connecting self‐reported caregiver distress to turnover intentions and absenteeism. Journal of Patient Safety, 17(3), 195–199. 10.1097/PTS.0000000000000301 27811593 PMC5413437

[jan16291-bib-0005] Burlison, J. D. , Scott, S. D. , Browne, E. K. , Sierra, G. , & Hoffman, J. M. (2017). The second victim experience and support tool: Validation of an organizational resource for assessing second victim effects and the quality of support resources. Journal of Patient Safety, 13(2), 93–102. 10.1097/PTS.0000000000000129.The 25162208 PMC4342309

[jan16291-bib-0006] Busch, I. M. , Moretti, F. , Campagna, I. , Benoni, R. , Tardivo, S. , Wu, A. W. , & Rimondini, M. (2021). Promoting the psychological well‐being of healthcare providers facing the burden of adverse events: A systematic review of second victim support resources. International Journal of Environmental Research and Public Health, 18(10), 5080. 10.3390/ijerph18105080 34064913 PMC8151650

[jan16291-bib-0007] Busch, I. M. , Moretti, F. , Purgato, M. , Barbui, C. , Wu, A. W. , & Rimondini, M. (2020a). Dealing with adverse events: A meta‐analysis on second Victims' coping strategies. Journal of Patient Safety, 16(2), E51–E60. 10.1097/PTS.0000000000000661 32168267

[jan16291-bib-0008] Busch, I. M. , Moretti, F. , Purgato, M. , Barbui, C. , Wu, A. W. , & Rimondini, M. (2020b). Psychological and psychosomatic symptoms of second victims of adverse events: A systematic review and meta‐analysis. Journal of Patient Safety, 16(2), E61–E74. 10.1097/PTS.0000000000000589 30921046 PMC7386870

[jan16291-bib-0009] Clarkson, M. D. , Haskell, H. , Hemmelgarn, C. , & Skolnik, P. J. (2019). Abandon the term “second victim”. BMJ (Online), 364, l1233. 10.1136/bmj.l1233 30917966

[jan16291-bib-0010] Coughlan, B. , Powell, D. , & Higgins, M. F. (2017). The second victim: A review. European Journal of Obstetrics, Gynecology, and Reproductive Biology, 213, 11–16. 10.1016/j.ejogrb.2017.04.002 28526169

[jan16291-bib-0011] Draus, C. , Mianecki, T. B. , Musgrove, H. , Bastien, D. J. , Greggs, D. , Halash, C. , Bellamy, C. , Lewis, A. , & MacKenzie, W. (2022). Perceptions of nurses who are second victims in a hospital setting. Journal of Nursing Care Quality, 37(2), 110–116. 10.1097/NCQ.0000000000000603 34775418

[jan16291-bib-0012] Edrees, H. , Connors, C. , Paine, L. , Norvell, M. , Taylor, H. , & Wu, A. W. (2016). Implementing the RISE second victim support programme at the Johns Hopkins Hospital: A case study. BMJ Open, 6(9), e011708. 10.1136/bmjopen-2016-011708 PMC5051469 27694486

[jan16291-bib-0013] Elliott, R. A. , Camacho, E. , Campbell, F. , Jankovic, D. , Martyn St James, M. , Kaltenthaler, E. , Wong, R. , Sculpher, M. J. , & Faria, R. (2018). Prevalence and economic burden of medication errors in the NHS in England: Rapid evidence synthesis and economic analysis of THE prevalence and burden of medication error. Universities of Sheffield and York. https://www.eepru.org.uk/wp‐content/uploads/2018/02/medication‐error‐report‐revised‐final.2‐22022018.pdf

[jan16291-bib-0014] Finney, R. E. , Czinski, S. , Fjerstad, K. , Arteaga, G. M. , Weaver, A. L. , Riggan, K. A. , Allyse, M. A. , Long, M. E. , Torbenson, V. E. , & Rivera‐Chiauzzi, E. Y. (2021). Evaluation of a second victim peer support program on perceptions of second victim experiences and supportive resources in pediatric clinical specialties using the second victim experience and support tool (SVEST). Journal of Pediatric Nursing, 61, 312–317. 10.1016/j.pedn.2021.08.023 34500175

[jan16291-bib-0015] Guo, Y. F. , Luo, Y. H. , Lam, L. , Cross, W. , Plummer, V. , & Zhang, J. P. (2018). Burnout and its association with resilience in nurses: A cross‐sectional study. Journal of Clinical Nursing, 27(1–2), 441–449. 10.1111/jocn.13952 28677270

[jan16291-bib-0016] Härkänen, M. , Vehviläinen‐Julkunen, K. , Murrells, T. , Rafferty, A. M. , & Franklin, B. D. (2018). Medication administration errors and mortality: Incidents reported in England and Wales between 2007 and 2016. Research in Social and Administrative Pharmacy, 15(7), 858–863. 10.1016/j.sapharm.2018.11.010 30528260

[jan16291-bib-0017] Järvisalo, P. , Haatainen, K. , Von Bonsdorff, M. , Turunen, H. , & Härkänen, M. (2023). Interventions to support nurses as second victims of patient safety incidents: A qualitative study of nurse managers' perceptions. Journal of Advanced Nursing, 80(6), 1–14. 10.1111/jan.16013 38071607

[jan16291-bib-0018] Kappes, M. , Delgado‐Hito, P. , Contreras, V. R. , & Romero‐García, M. (2023). Prevalence of the second victim phenomenon among intensive care unit nurses and the support provided by their organizations. Nursing in Critical Care, 28(6), 1022–1030. 10.1111/nicc.12967 37614030

[jan16291-bib-0019] Kappes, M. , Romero‐García, M. , & Delgado‐Hito, P. (2021). Coping strategies in health care providers as second victims: A systematic review. International Nursing Review, 68(4), 471–481. 10.1111/inr.12694 34118061

[jan16291-bib-0020] Krommer, E. , Ablöscher, M. , Klemm, V. , Gatterer, C. , Rösner, H. , Strametz, R. , Huf, W. , & Ettl, B. (2023). Second victim phenomenon in an Austrian hospital before the implementation of the systematic collegial help program KoHi: A descriptive study. International Journal of Environmental Research and Public Health, 20(3), 1913. 10.3390/ijerph20031913 36767279 PMC9915153

[jan16291-bib-0021] Liukka, M. , Steven, A. , Moreno, M. F. V. , Sara‐Aho, A. M. , Khakurel, J. , Pearson, P. , Turunen, H. , & Tella, S. (2020). Action after adverse events in healthcare: An integrative literature review. International Journal of Environmental Research and Public Health, 17(13), 1–18. 10.3390/ijerph17134717 PMC7369881 32630041

[jan16291-bib-0022] MacKinnon, D. P. , Lockwood, C. M. , & Williams, J. (2004). Confidence limits for the indirect effect: Distribution of the product and resampling Methods. Multivariate Behavioral Research, 39(1), 99–128. 10.1207/s15327906mbr3901_4 20157642 PMC2821115

[jan16291-bib-0023] Mahat, S. , Rafferty, A. M. , Vehviläinen‐Julkunen, K. , & Härkänen, M. (2022). Negative emotions experienced by healthcare staff following medication administration errors: A descriptive study using text‐mining and content analysis of incident data. BMC Health Services Research, 22(1), 1–11. 10.1186/s12913-022-08818-1 36463187 PMC9719256

[jan16291-bib-0024] Mathebula, L. C. , Filmalter, C. J. , Jordaan, J. , & Heyns, T. (2022). Second victim experiences of healthcare providers after adverse events: A cross‐sectional study. Health SA Gesondheid, 27, 1–6. 10.4102/hsag.v27i0.1858 PMC9453125 36090235

[jan16291-bib-0025] Mira, J. J. , Carrillo, I. , Lorenzo, S. , Ferrús, L. , Silvestre, C. , Pérez‐Pérez, P. , Olivera, G. , Iglesias, F. , Zavala, E. , Maderuelo‐Fernández, J. Á. , Vitaller, J. , Nuño‐Solinís, R. , & Astier, P. (2015). The aftermath of adverse events in spanish primary care and hospital health professionals. BMC Health Services Research, 15(1), 1–9. 10.1186/s12913-015-0790-7 25886369 PMC4394595

[jan16291-bib-0026] Mohd Kamaruzaman, A. Z. , Ibrahim, M. I. , Mokhtar, A. M. , Mohd Zain, M. , Satiman, S. N. , & Yaacob, N. M. (2022a). The effect of second‐victim‐related distress and support on work‐related outcomes in tertiary care hospitals in Kelantan, Malaysia. International Journal of Environmental Research and Public Health, 19(11), 6454. 10.3390/ijerph19116454 35682042 PMC9180130

[jan16291-bib-0027] Mohd Kamaruzaman, A. Z. , Ibrahim, M. I. , Mokhtar, A. M. , Mohd Zain, M. , Satiman, S. N. , & Yaacob, N. M. (2022b). Translation and Validation of the Malay Revised Second Victim Experience and Support Tool (M‐SVEST‐R) among Healthcare Workers in Kelantan, Malaysia. International Journal of Environmental Research and Public Health, 19(2045), 1–16. 10.3390/ijerph19042045 PMC8872429 35206235

[jan16291-bib-0028] Mok, W. Q. , Chin, G. F. , Yap, S. F. , & Wang, W. (2020). A cross‐sectional survey on nurses' second victim experience and quality of support resources in Singapore. Journal of Nursing Management, 28(2), 286–293. 10.1111/jonm.12920 31789437

[jan16291-bib-0029] Mokline, B. , Anis, M. , & Abdallah, B. (2021). Individual resilience in the Organization in the Face of crisis: Study of the concept in the context of COVID‐19. Global Journal of Flexible Systems Management, 22, 219–231. 10.1007/s40171-021-00273-x 38624875 PMC8217778

[jan16291-bib-0030] Panagioti, M. , Khan, K. , Keers, R. N. , Abuzour, A. , Phipps, D. , Kontopantelis, E. , Bower, P. , Campbell, S. , Haneef, R. , Avery, A. J. , & Ashcroft, D. M. (2019). Prevalence, severity, and nature of preventable patient harm across medical care settings: Systematic review and meta‐analysis. BMJ, 366(I4185), l4185. 10.1136/bmj.l4185 31315828 PMC6939648

[jan16291-bib-0031] Panella, M. , Rinaldi, C. , Leigheb, F. , Donnarumma, C. , Kul, S. , Vanhaecht, K. , & Di Stanislao, F. (2016). The determinants of defensive medicine in Italian hospitals: The impact of being a second victim. Revista de Calidad Asistencial : Organo de La Sociedad Espanola de Calidad Asistencial, 31(Suppl 2), 20–25. 10.1016/j.cali.2016.04.010 27373579

[jan16291-bib-0032] Parks‐Savage, A. , Archer, L. , Newton, H. , Wheeler, E. , & Huband, S. R. (2018). Prevention of medical errors and malpractice: Is creating resilience in physicians part of the answer? International Journal of Law and Psychiatry, 60, 35–39. 10.1016/j.ijlp.2018.07.003 30217328

[jan16291-bib-0033] Preacher, K. J. , & Hayes, A. F. (2004). SPSS and SAS procedures for estimating indirect effects in simple mediation models. Behavior Research Methods, Instruments, & Computers, 36(4), 717–731. 10.1002/jcp.28952 15641418

[jan16291-bib-0034] Preacher, K. J. , & Hayes, A. F. (2008). Asymptotic and resampling strategies for assessing and comparing indirect effects in multiple mediator models. Behavior Research Methods, 40(3), 879–891. 10.3758/BRM.40.3.879 18697684

[jan16291-bib-0035] Rivera‐Chiauzzi, E. , Finney, R. E. , Riggan, K. A. , Weaver, A. L. , Long, M. E. , Torbenson, V. E. , & Allyse, M. A. (2022). Understanding the second victim experience among multidisciplinary providers in obstetrics and gynecology. Journal of Patient Safety, 18(2), E463–E469. 10.1097/PTS.0000000000000850 33871416 PMC8521555

[jan16291-bib-0036] Robertson, J. J. , & Long, B. (2019). Medicine's shame problem. Journal of Emergency Medicine, 57(3), 329–338. 10.1016/j.jemermed.2019.06.034 31431319

[jan16291-bib-0037] Scott, S. D. , Hirschinger, L. E. , Cox, K. R. , McCoig, M. , Brandt, J. , & Hall, L. W. (2009). The natural history of recovery for the healthcare provider “second victim” after adverse patient events. Quality & Safety in Health Care, 18(5), 325–330. 10.1136/qshc.2009.032870 19812092

[jan16291-bib-0038] Scott, S. D. , Hirschinger, L. E. , Cox, K. R. , McCoig, M. , Hahn‐Cover, K. , Epperly, K. M. , Phillips, E. C. , & Hall, L. W. (2010). Caring for our own: Deploying a systemwide second victim rapid response team. Joint Commission Journal on Quality and Patient Safety, 36(5), 233–240. 10.1016/S1553-7250(10)36038-7 20480757

[jan16291-bib-0039] Seys, D. , Wu, A. W. , Van Gerven, E. , Vleugels, A. , Euwema, M. , Panella, M. , Scott, S. D. , Conway, J. , Sermeus, W. , & Vanhaecht, K. (2013). Health care professionals as second victims after adverse events: A systematic review. Evaluation & the Health Professions, 36(2), 135–162. 10.1177/0163278712458918 22976126

[jan16291-bib-0040] Shapiro, J. , Whittemore, A. , & Tsen, L. C. (2014). Instituting a culture of professionalism: The establishment of a Center for Professionalism and Peer Support. *The* . Joint Commission Journal on Quality and Patient Safety, 40(4), 168‐AP1. 10.1016/S1553-7250(14)40022-9 24864525

[jan16291-bib-0041] Sheikhrabori, A. , Peyrovi, H. , & Hamidreza, K. (2022). The Main features of resilience in health care providers : A scoping review. Medical Journal of the Islamic Republic of Iran, 36(3), 22–37. 10.47176/mjiri.36.3 35999934 PMC9386779

[jan16291-bib-0042] Strametz, R. , Fendel, J. C. , Koch, P. , Roesner, H. , Zilezinski, M. , Bushuven, S. , & Raspe, M. (2021). Prevalence of second victims, risk factors, and support strategies among german nurses (Sevid‐ii survey). International Journal of Environmental Research and Public Health, 18(20), 1–15. 10.3390/ijerph182010594 PMC8535996 34682342

[jan16291-bib-0043] Strametz, R. , Koch, P. , Vogelgesang, A. , Burbridge, A. , Rösner, H. , Abloescher, M. , Huf, W. , Ettl, B. , & Raspe, M. (2021). Prevalence of second victims, risk factors and support strategies among young German physicians in internal medicine (SeViD‐I survey). Journal of Occupational Medicine and Toxicology, 16, 1–11. 10.1186/s12995-021-00300-8 33781278 PMC8005860

[jan16291-bib-0044] Strametz, R. , Siebold, B. , Heistermann, P. , Haller, S. , & Bushuven, S. (2022). Validation of the German version of the second victim experience and support tool—Revised. Journal of Patient Safety, 18(3), 182–192. 10.1097/PTS.0000000000000886 34387250 PMC9359781

[jan16291-bib-0045] Tumelty, M. E. (2021). The second victim: A contested term? Journal of Patient Safety, 17(8), E1488–E1493. 10.1097/PTS.0000000000000558 30570536

[jan16291-bib-0046] Ullström, S. , Sachs, M. A. , Hansson, J. , Øvretveit, J. , & Brommels, M. (2014). Suffering in silence: A qualitative study of second victims of adverse events. BMJ Quality and Safety, 23(4), 325–331. 10.1136/bmjqs-2013-002035 PMC3963543 24239992

[jan16291-bib-0047] Van Gerven, E. , Seys, D. , Panella, M. , Sermeus, W. , Euwema, M. , Federico, F. , Kenney, L. , & Vanhaect, K. (2014). Involvement of health‐care professionals in an adverse event: The role of management in supporting their workforce. Polish Archives of Internal Medicine‐Polskie Archiwum Medycyny Wewnetrznej, 124(6), 313–320. 10.20452/pamw.2297 24781784

[jan16291-bib-0048] von Elm, E. , Altman, D. G. , Egger, M. , Pocock, S. J. , Gøtzsche, P. C. , & Vandenbroucke, J. P. (2007). The strengthening the reporting of observational studies in epidemiology (STROBE) statement: Guidelines for reporting observational studies. Lancet (London, England), 370(9596), 1453–1457. 10.1016/S0140-6736(07)61602-X 18064739

[jan16291-bib-0049] White, R. M. , & Delacroix, R. (2020). Second victim phenomenon: Is ‘just culture’ a reality? An integrative review. Applied Nursing Research, 56(September 2018), 151319. 10.1016/j.apnr.2020.151319 32868148

[jan16291-bib-0050] Winning, A. M. , Merandi, J. , Rausch, J. R. , Liao, N. , Hoffman, J. M. , Burlison, J. D. , & Gerhardt, C. A. (2021). Validation of the second victim experience and support tool‐revised in the neonatal intensive care unit. Journal of Patient Safety, 17(8), 531–540. 10.1097/PTS.0000000000000659 32175958

[jan16291-bib-0051] World Health Organization . (2024a). Fair share for health and care: gender and the undervaluation of health and care work . https://iris.who.int/bitstream/handle/10665/376191/9789240082854‐eng.pdf?sequence=1

[jan16291-bib-0052] World Health Organization . (2024b). Process of translation and adaptation of instruments . https://www.who.int/substance_abuse/research_tools/translation/en/

[jan16291-bib-0053] Wu, A. (2000). Medical error: The second victim. BMJ, 320(7237), 726–727. 10.1136/bmj.320.7237.726 10720336 PMC1117748

[jan16291-bib-0054] Wu, A. , Shapiro, J. , Harrison, R. , Scott, S. D. , Connors, C. , Kenney, L. , & Vanhaecht, K. (2020). The impact of adverse events on clinicians: What's in a name? Journal of Patient Safety, 16(1), 65–72. 10.1097/PTS.0000000000000256 29112025

[jan16291-bib-0055] Xu, H. , Cao, X. , Jin, Q. X. , Wang, R. S. , Zhang, Y. H. , & Chen, Z. H. (2022). Distress, support and psychological resilience of psychiatric nurses as second victims after violence: A cross‐sectional study. Journal of Nursing Management, 30(6), 1777–1787. 10.1111/jonm.13711 35689407

[jan16291-bib-0056] Zhang, X. , Li, Q. , Guo, Y. , & Lee, S. Y. (2019). From organisational support to second victim‐related distress: Role of patient safety culture. Journal of Nursing Management, 27(8), 1818–1825. 10.1111/jonm.12881 31556205

